# Heightened Stress Reactivity in Response to an Attachment Related Stressor in Patients With Medically Treated Primary Hypertension

**DOI:** 10.3389/fpsyt.2021.718919

**Published:** 2021-09-09

**Authors:** Elisabeth M. Balint, Marc N. Jarczok, Dominik Langgartner, Stefan O. Reber, Simon Endes, Arno Schmidt-Trucksäss, Alexandra Funk, Julia Klinghammer, Susanne Campbell, Harald Gündel, Christiane Waller

**Affiliations:** ^1^Ulm University Medical Center, Department of Psychosomatic Medicine and Psychotherapy, Ulm, Germany; ^2^Laboratory for Molecular Psychosomatics, Department of Psychosomatic Medicine and Psychotherapy, University Ulm, Ulm, Germany; ^3^Division of Sports and Exercise Medicine, Department of Sport, Exercise and Health, University of Basel, Basel, Switzerland; ^4^Department of Psychosomatic Medicine and Psychotherapy, Nuremberg General Hospital, Paracelsus Medical Private University, Nuremberg, Germany

**Keywords:** autonomic nervous system, hypothalamic pituitary adrenal axis, attachment, mental stress, antihypertensive treatment

## Abstract

**Background:** A heightened stress reactivity to mental stress tasks has been shown in hypertensive patients and might contribute to a higher disease risk. We investigated this hyperreactivity with regard to an attachment related stressor that focuses on emotions instead of performance and we examined whether this effect can also be found in patients on antihypertensive drugs.

**Materials and Methods:** Fifty patients with primary hypertension, treated with at least one antihypertensive drug, were compared with 25 healthy individuals. After 10 min of rest, they participated in an attachment-related interview (Adult Attachment Projective picture system, AAP) and were exposed to an attachment-related stressor (Separation Recall, SR), a short-time stressor which activates attachment-related emotions and thoughts by talking 5 min about a personal experience of loneliness. Blood samples to measure adrenocorticotrope hormone (ACTH), cortisol, norepinephrine, epinephrine, and dopamine were taken. Blood pressure, heart rate and arterial stiffness were measured at rest, after AAP, after SR and 10 min after recovery. Standard deviation of normal-to-normal intervals (SDNN) and root mean square of successive differences (RMSSD) were calculated. Parameters were compared using Mann Whitney *U*-test and linear mixed-effects regression models controlling for age and body mass index (BMI) after logarithmic transformation if appropriate.

**Results:** Healthy test persons were younger and had lower BMI than patients. Comparing the two groups there were no significant differences in blood pressure and heart rate at rest. Both stressors provoked a significant response in almost all parameters. Results of the post-estimation of contrasts from linear mixed-effects regression models showed a steeper rise in systolic BP and arterial stiffness as well as a more pronounced decline in SDNN in hypertensive patients than in healthy controls. Levels of cortisol rose earlier and higher in hypertensive patients than in healthy controls.

**Conclusion:** Vascular, autonomic, and hypothalamic pituitary adrenal axis response is heightened in medicated subjects with hypertension in response to attachment-focused stressors compared to healthy subjects. We conclude that the remaining hyper-reactivity even with sufficient antihypertensive medication still poses a substantial risk for affected patients. New ways to diminish this risk should be developed.

## Introduction

One out of three individuals is affected by hypertension in an industrial country like Germany (1). It is estimated to account for 7.6 million pre-mature deaths worldwide, and about 54% of strokes and 47% of ischemic cardiac diseases are a direct consequence of hypertension (2). Hence, it is a main risk factor for ischemic disease and death. In contrary to this and to decades of research, a breakthrough in prevention and therapy is not yet achieved. The amount of controlled hypertension is still only about 50% (1).

Higher resting blood pressure (BP) is correlated to higher risk of cardiovascular (CV) disease (3). Beyond resting BP, BP variability is a recognized risk factor (4). The response to stressors is of special interest in hypertension research. In normotensives, BP reactivity is predictive for the development of hypertension, and this has been shown for physical (5) as well as for mental stress (6–8). Also, sympathoadrenal stress reactivity predicts future BP (9). Cortisol responses to mental stress are as well-correlated with the incidence of hypertension (10). Stress response seems to be generally higher in individuals with systemic hypertension, not only in terms of a higher BP response, but also with regard to norepinephrine excretion and cortisol (11, 12). Overall, an enhanced CV response to mental stressors is associated with incident hypertension (13).

Existing studies are almost exclusively covering hypertensive patients without medication. Nevertheless, about one third of individuals with hypertension take antihypertensive medication (1). The results might differ from unmedicated subjects, as medication like beta-blocking agents should diminish the sympathetic hyperreactivity and drugs like angiotensin converting enzyme (ACE) inhibitors and AT1 receptor antagonists are found to reduce levels of adrenocorticotropic hormone (ACTH) and cortisol (14). This is important, as the goal of medication is not merely to reduce BP, but to prevent further disease like myocardial infarction (MI). CV deaths can be triggered by stress (15), and a heightened cortisol response to mental stress is associated with the progression of coronary artery calcification (16). Therefore, it is important to know whether medicated individuals suffering from hypertension still show a hyper-reactive stress response.

Mental stressors commonly used for evoking a stress response are cognitive and performance-oriented like the mental arithmetic. The Trier Social Stress Test also includes a social component implementing a job interview (17). The majority of hypertensive patients is older than 65 years and has already retired. Thus, this kind of performance-focused stressor seems to be inappropriate. Loneliness is a major risk factor for mortality (18), so we sought a stressor associated with (missing) interpersonal contact. This kind of stressor is the separation recall (SR) (19), which has been evaluated against mental arithmetic and has proven to be effective. Remembering and talking about a situation of loneliness and abandonment in one's own life triggers the memories and emotions linked to this situation. In order to broaden the context from loneliness and being abandoned to other attachment-related topics, we added an attachment-related interview, which is called adult attachment projective (AAP) (20). Nine different scenes of people being alone or with another person serve as stimuli for individual narratives and hereby activate the individual attachment patterns.

The present study aims at evaluating whether medicated patients with hypertension still show a heightened stress response to attachment/interpersonal-focused stressors.

## Materials and Methods

### Study Population

Fifty hypertensive patients with primary hypertension, treated with at least one antihypertensive drug, were recruited from the Cardiologic Outpatient Clinic at the Ulm University Hospital. The following inclusion criteria were applied: age between 18 and 80 years and sufficient knowledge of the German language. Patients with heart insufficiency with ejection fraction <35%, severe valvular stenosis or insufficiency, end-stage renal disease with regular dialysis, current alcohol or drug abuse, chronic rheumatic diseases, obvious cognitive deficits following stroke, current psychosis and dementia, cortisol intake due to other comorbidities during the last 3 months, and those who received renal denervation for treatment of uncontrolled hypertension were excluded from the study.

Healthy participants were recruited by advertisement in the local newspaper. They were questioned by telephone about existing diseases and medication, those being criteria to be excluded from this study. People with asymptomatic substituted hypothyreosis were not excluded. We checked known diagnosis and medication again immediately before the stress test. Out of the initially recruited 31 healthy controls, one person took Trimipramin (Stangyl®, Sanofi-Aventis, Germany), one suffered from diabetes (newly diagnosed), two were on cortisol medication (topic/inhalative), and two had arterial hypertension (newly diagnosed). These individuals were excluded, leaving 25 individuals as healthy controls.

### Procedures

**Figure 1:**
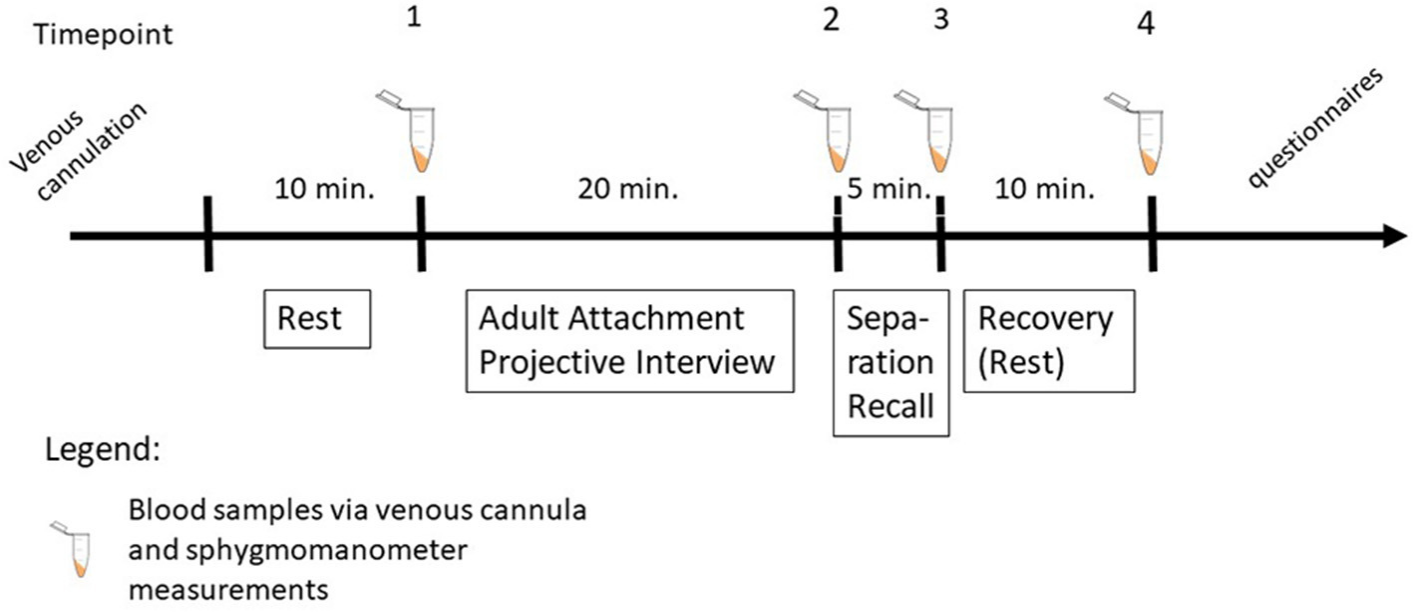
Schematic overview of the experiment.

The study protocol was approved by the ethical review board of Ulm University (no. 129/12) in accordance to the Helsinki Declaration. All subjects gave written informed consent. All subjects were instructed neither to smoke nor to drink coffee or black tea and to have lunch prior to 12 a.m. on the day of measurement. An overview of the experiment is shown in Figure 1. After arrival at the lab at 02:00 p.m., medication and medical history were assessed and a venous cannula was placed. Electrodes and BP cuffs were attached to the chest, both upper arms and both ankles, respectively. BP, brachial-ankle pulse wave velocity (baPWV), central PWV (cPWV) and central pulse pressure (cPP) were automatically measured at different points of time using an oscillometric sphygmomanometer (Vasera VS-1500N, serial number 50000071, Fukuda Denshi Co ltd., Tokyo, Japan) on the right and the left upper arm and ankles as described below, while ECG was recorded continuously using NeXus-10 wireless physiological monitoring (serial number 0928100209, Mindmedia, Oldenzaal, Netherlands). In order to minimize artifacts via arm or leg movements, the whole procedure was conducted while the patient was lying. After 10 min of rest, an attachment-related interview (AAP) (20) and an attachment-related stressor (SR) (19) were performed. A short-time stressor which activates attachment-related emotions and thoughts by talking 5 min about a personal experience of loneliness. Blood samples were taken and sphygmomanometer measurements were performed after the AAP, the SR and 10 min of recovery. Mean HR was calculated for the penultimate minute of each time period (rest, AAP, SR, recovery). BP was calculated on the basis of the mean values of right and left arm measurement. Resting BP was calculated on the basis of the average of the two consecutive measurements.

The questionnaires described in the following section were completed after the measurement.

During SR three participants (two hypertensive, one healthy) were unable to identify an event when they felt lonely. Thus, no measurement results are available for them at time point 3.

### Questionnaires

Physical activity was measured using the Freiburg Questionnaire of Physical Activity (21). The questions serve to specify physical activity during daily life in minutes per week, e.g., walking to the work place as well as doing sports. By applying metabolic equivalent of task (MET) estimates for every activity, a MET sum score can be calculated. We categorized the sum score in <500, 500–999, 1,000–1,499, and >1,500 MET-min/week according to Jeong (22).

Depressive and anxious symptoms were assessed using the German version of the Hospital Anxiety and Depression Scale (HADS-D, HADS-A) (23, 24). Seven items on each scale are rated on a three-point Likert scale and summed up, resulting in a range of 0–21. Higher scores indicate greater severity of symptoms.

### Heart Rate Variability

HRV parameters were calculated using Kubios HRV Premium 3.3.1 (Kubios Oy, Kuopio, Finland). We chose to calculate HRV from a short time window of 60 s, preferably free of artifacts (3), which was available in most measurements. The 60-s-window was placed in the last of the 5 min prior to the four measurement time points and was manually screened for artifacts. If artifacts were present, the window was moved to find an artifact-free (preferably) period inside the 5-min-window. The maximum number of allowed artifact per minute was one. In addition to the manual screening and correction of artifacts, the automated artifact correction of Kubios, set to a low threshold, was applied. Five data sets could not be included due to technical reasons (missing data, corrupt data). Four individuals with hypertension had no constant sinus rhythm (2x atrial fibrillation, 1x trigeminus, 1x bigeminus), so the number of ECG valid for HRV calculation was 66 (41 hypertensives, 25 healthy). Of the included data, six measurements (9%) had one artifact per minute.

We calculated the time-domain parameters SDNN (standard deviation of normal-to-normal intervals) and RMSSD (root mean square of successive differences) as they can be calculated from a short time window of 1 min, which is not recommended for frequency-domain parameters like the low frequency band (25). SDNN reflects the global activity of the autonomic nervous system (ANS) and RMSSD represents the rapid changes in HR caused by the parasympathetic branch of the ANS (25).

### Blood Parameters

Venous cannulation was not successful in three hypertensive patients. In another three patients, blood could not be drawn from the cannula at later time points. We did not place a new cannula two avoid additional stressful stimuli during the experiment.

Blood samples were drawn from the venous cannula using a tube with coagulation activator (S-Monovette Serum, Sarstedt, Nümbrecht, Germany) for ACTH and a tube containing EDTA for the other blood parameters (S-Monovette EDTA, Sarstedt, Nümbrecht, Germany). The tubes were cooled directly before use and centrifuged immediately with 2,000 G and 3,570 U for 10 min (ACTH) and for 5 min (other blood parameters), respectively. Blood samples were frozen at −80 degree. Catecholamines (norepinephrine, epinephrine, dopamine) were measured using High Pressure Liquid Chromatography (CLC300, Chromsystems, Munich, Germany) and electrochemic detection (CLC100, Chromsystems, Munich, Germany). Plasma samples were analyzed using commercially available ELISA kits for ACTH (analytical sensitivity <1 pg/ml, intra-assay and inter-assay coefficients of variation <8.8%, IBL International, Hamburg, Germany) and Cortisol (analytical sensitivity <2.46 ng/ml, intraassay and inter-assay coefficients of variation <3.5%, IBL International, Hamburg, Germany).

### Statistics

Parameters were compared using the non-parametric Mann-Whitney-*U*-test as not all descriptive parameters were normally distributed and Chi-square test (Table 1) where appropriate (SPSS Statistics 25, IBM, USA). A linear mixed-effects model with random intercepts were fitted using (STATA 15.1, STATA Corp, USA). Dependent variables were SBP, DBP, HR, log(SDNN), log(RMSSD), log(ACTH), log(Cortisol), log(Norepinephrine), log(Epinephrine), log(Dopamine), baPWV, cPWV, log(cPP). Time was level 1, the individual level (Persons) was on level 2. A two-way-interaction between group (Healthy vs. Hypertensive) and time (rest [1], AAP [2], SR [3], recovery [4]) was modeled using restricted maximum likelihood. Regression parameters were normalized choosing the function that best approximates Gaussian/normal distribution as marked. Age and BMI differed significantly between hypertensives and healthy individuals and therefore were included in the models as covariates in the fixed effect part. Post-estimates from each model were contrasts of marginal linear predictions to test the effects of group, time, and their interaction (Table 2). A significance level of *p* < 0.05 was regarded significant. Marginal mean plots were calculated (**Figures 3**–**7**) at average at fixed values for group and time interaction and averaging over the remaining covariates age and BMI.

**Table 1:** Study population.

	**Hypertensive patients** ***N*** **= 50**		**Healthy controls** ***N*** **= 25**		
	**Median/*N***	**Interquartile range/%**	**Median/*N***	**Interquartile range/%**	***P*-value**
Age [years]	66	57–72	61	54–65	0.018
Sex (male)	38	76%	14	56%	0.111
In partnership	41	85%	19	76%	0.347
Higher education	12	24%	10	4%	0.183
Physical activity >500 MET-min/week	43	86%	23	92%	0.768
BMI [kg/m^2^]	28.1	26.3–31.3	24.3	22.7–27.4	<0.001
HADS anxious symptoms	6	2–8	6	1.5–7	0.566
HADS severe anxious symptoms	4	8%	0	0	0.590
HADS depressive symptoms	4	2–8	3	1.5–5	0.130
HADS severe depressive symptoms	6	12%	0	0	0.354
Systolic blood pressure [mmhg] (rest)	133.0	127.3–142.1	129	121.5–140.0	0.222
Diastolic blood pressure [mmhg] (rest)	78.8	75.8–83.6	79.5	73.0–88.0	0.800
Heart rate [bpm] (rest)	60.4	56.2–67.8	59.6	57.0–65.0	0.783
Number of diagnoses	6	4–7	0	0	
Number of medication	7	5–9	0	0	
Number of antihypertensive medication	3	2–4	0	0	
Coronary artery disease	37	74%	0	0	
History of myocardial infarction	20	40%	0	0	
Obesity (BMI>30 kg/m^2^)	19	38%	0	0	
Diabetes	13	26%	0	0	
Smoking	4	8%	0	0	
Medication:					
ACE Inhibitor/AT1 Receptor Antagonist (mostly Ramipril/Candesartan)	48	96%	0	0	
Beta-blocking agent (mostly Metoprolol)	39	78%	0	0	
Diuretics (mostly Hydrochlorothiazid)	25	50%	0	0	
Calcium channel blocker (mostly Amlodipin)	24	48%	0	0	

*P-values calculated with Mann Whitney U-test for metric and chi square test for dichotomous variables*.

**Table 2:** Post-estimation of contrasts from linear mixed-effects regression model for stress parameter reactivity.

**Dependent variables**	**Group (** ***df*** **= 1)**		**Time (** ***df*** **= 3)**		Group*time ( ***df*** **= 3)**	
	**chi2**	***p***	**chi2**	***p***	**chi2**	***p***
Systolic blood pressure	0.15	0.703	171.89	<0.001	8.80	0.032
Diastolic blood pressure	0.05	0.819	149.83	<0.001	5.92	0.116
Heart rate	0.00	0.993	260.04	<0.001	7.69	0.053
log(SDNN)	1.69	0.194	91.81	<0.001	18.05	<0.001
log(RMSSD)	0.69	0.406	82.10	<0.001	4.25	0.236
log(ACTH)	1.25	0.265	3.57	0.312	4.98	0.173
log(Cortisol)	7.48	0.006	38.66	<0.001	42.76	<0.001
log(Dopamine)	15.50	<0.001	2.61	0.455	3.95	0.267
log(Norepinephrine)	1.87	0.172	21.17	<0.001	2.40	0.493
log(Epinephrine)	4.04	0.045	2.36	0.502	0.27	0.965
baPWV	0.30	0.585	34.49	<0.001	2.84	0.418
cPWV	0.38	0.540	67.02	<0.001	5.83	0.120
log(cPP)	1.43	0.232	18.43	<0.001	1.39	0.709

*Post-estimation of contrasts from linear mixed-effects regression model with random intercepts*.

*Covariates included in the models: age, body mass index*.

*Time was level 1, the individual level (Persons) were on level 2*.

*A two-way-interaction between group (Healthy vs. Hypertensive) and time (rest [1], AAP [2], sSRr [3], recovery [4]) was modeled using restricted maximum likelihood*.

*Significance level: p < 0.05*.

*SDNN, standard deviation of normal to normal R-R intervals; RMSSD, root mean square of successive differences; ACTH, adreno-corticotrope hormone; baPWV, brachial-ankle pulse wave velocity; cPWV, central pulse wave velocity; cPP, central pulse pressure; df, degrees of freedom*.

## Results

### Study Population

Characteristics of the study population are shown in Table 1. Median age of hypertensive was 66 years (interquartile range: 57–72 years), while healthy controls were significantly younger (median age 61 years, interquartile range 54–65 years). A total of 76% of the hypertensives and 56% of the controls were male (difference insignificant). 85 and 76% were living in partnership, respectively. 86% of the hypertensive patients and 92% of controls reported physical activity of at least 500 MET-min/week, hereby meeting the minimum target of recommendations (26). Severe depressive or anxious symptoms were rare and only found in four (severe depressive symptoms), respectively six (severe anxious symptoms) hypertensive patients and in none of the control subjects. Hypertensive patients had higher BMI scores (median 28.1 kg/m^2^, interquartile range 26.3–31.3 kg/m^2^) than healthy controls (median 24.3 kg/m^2^, interquartile range 22.7–27.4 kg/m^2^). There were no significant differences in SBP, DBP, and HR at rest between the two groups.

**Figure 2:**
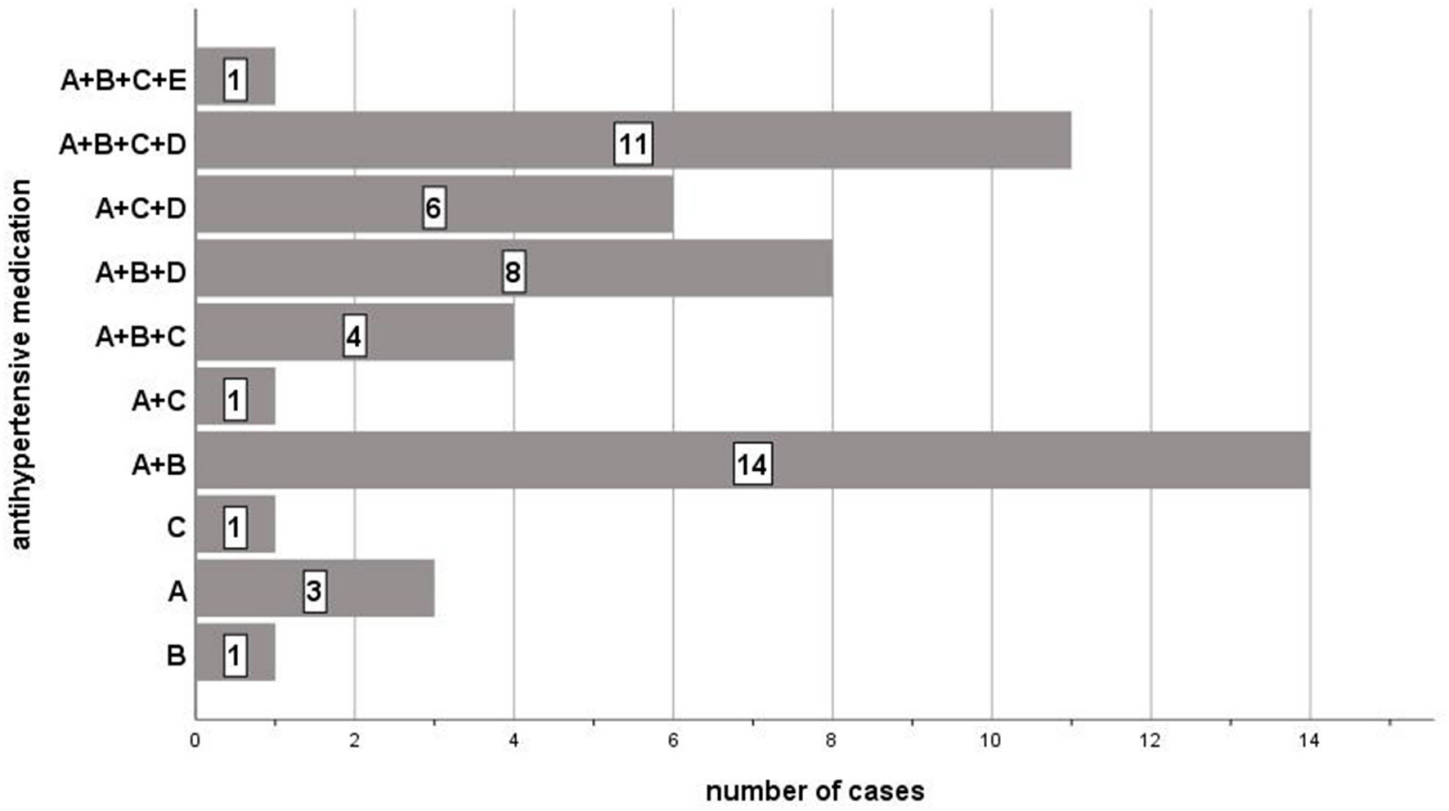
List of antihypertensive drugs and their combination taken by hypertensive study participants. A: ACE Inhibitor/AT1 Receptor Antagonist; B: Beta-Blocker; C: Calcium Channel Blocker; D: Diuretics; E: Alpha Blocker.

74% of hypertensive patients had already developed CAD. The median number of known diagnoses was 6 (interquartile range 4–7) and the mean number of drugs taken was 7 (interquartile range 5–9). Diabetes was prevalent in every fourth of the patients. Most of them took two (*N* = 15; 30%) or three (*N* = 16; 32%) antihypertensive drugs which were mostly ACE inhibitors or AT1 receptor antagonists (*N* = 48; 96%), followed by beta-blocking agents (*N* = 39; 78%). The antihypertensive medication is shown in detail in Figure 2.

### Blood Pressure

**Figure 3:**
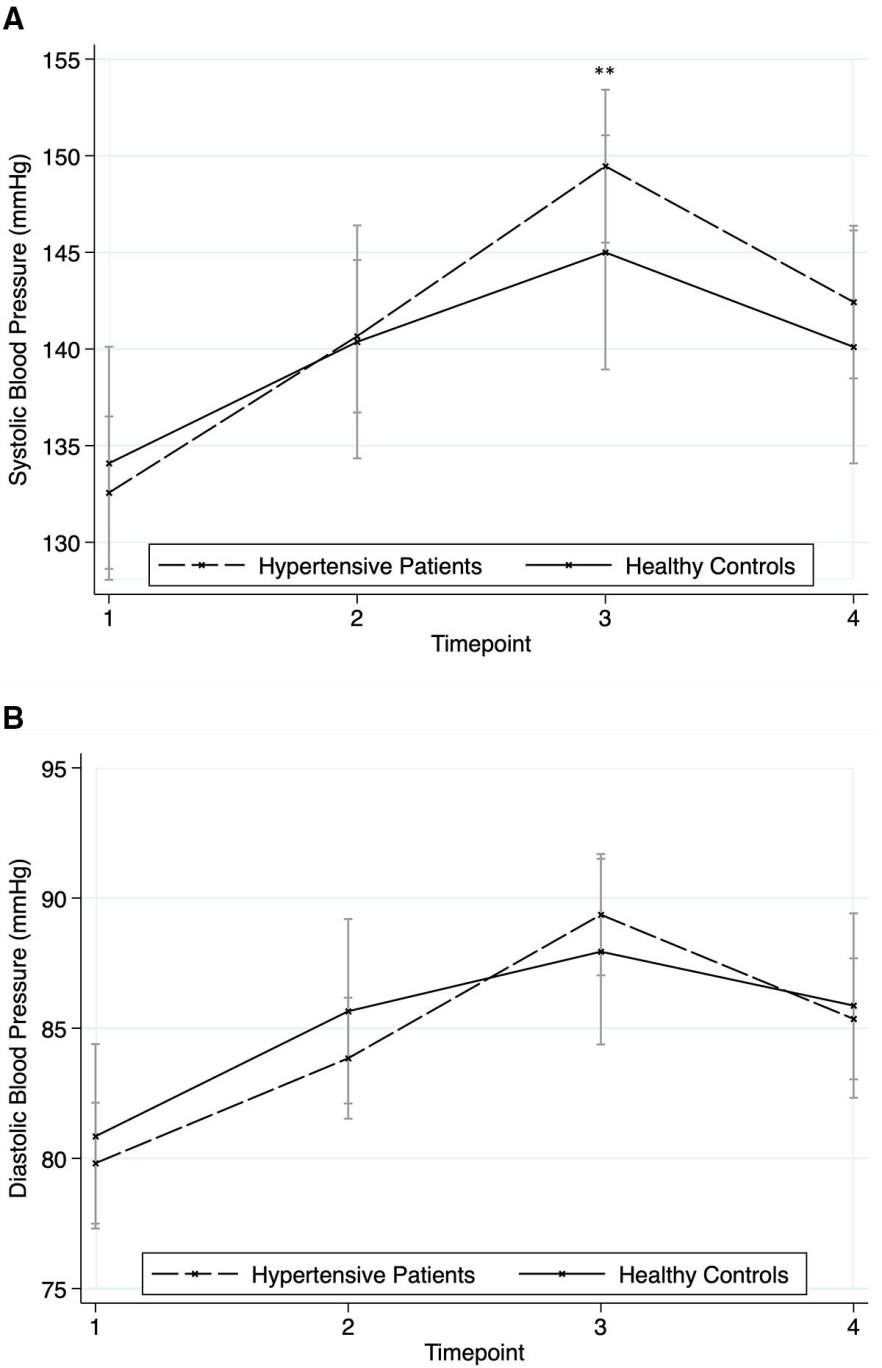
Responses in blood pressure to the stressors for hypertensive patients and healthy controls (mean ± SD). Timepoint 1: rest; timepoint 2: Attachment Interview (Adult Attachment Projective Picture System); timepoint 3: Separation Recall; timepoint 4: recovery. **(A)** Systolic Blood Pressure. **(B)** Diastolic Blood Pressure. Asterisks indicate significant group*time differences related to healthy controls at reference timepoint 1 (**p < 0.01).

Table 2 contains the results of post-estimation of contrasts from linear mixed-effects regression models for stress parameter reactivity. Complete regression tables are shown in Supplementary Material 1. Figure 3 shows the stress response of hypertensive patients and healthy controls for SBP (Figure 3A) and DBP (Figure 3B). Linear mixed-effects regression models for SBP showed a significant time effect (chi2 = 171.89, *p* < 0.001), no group effect (chi2 = 0.15, *p* = 0.703) and a significant group^*^time-effect (chi2 = 8.80, *p* = 0.032) with hypertensive patients showing significantly higher values at time point three (*z* = −2.81, *p* = 0.005). For DBP, only a significant time effect (chi2 = 149.83, *p* < 0.001) was found, while group (chi2 = 0.05, *p* = 0.819) and group^*^time-effect (chi2 = 5.92, *p* = 0.116) were not significant.

### Heart Rate and Heart Rate Variability

**Figure 4:**
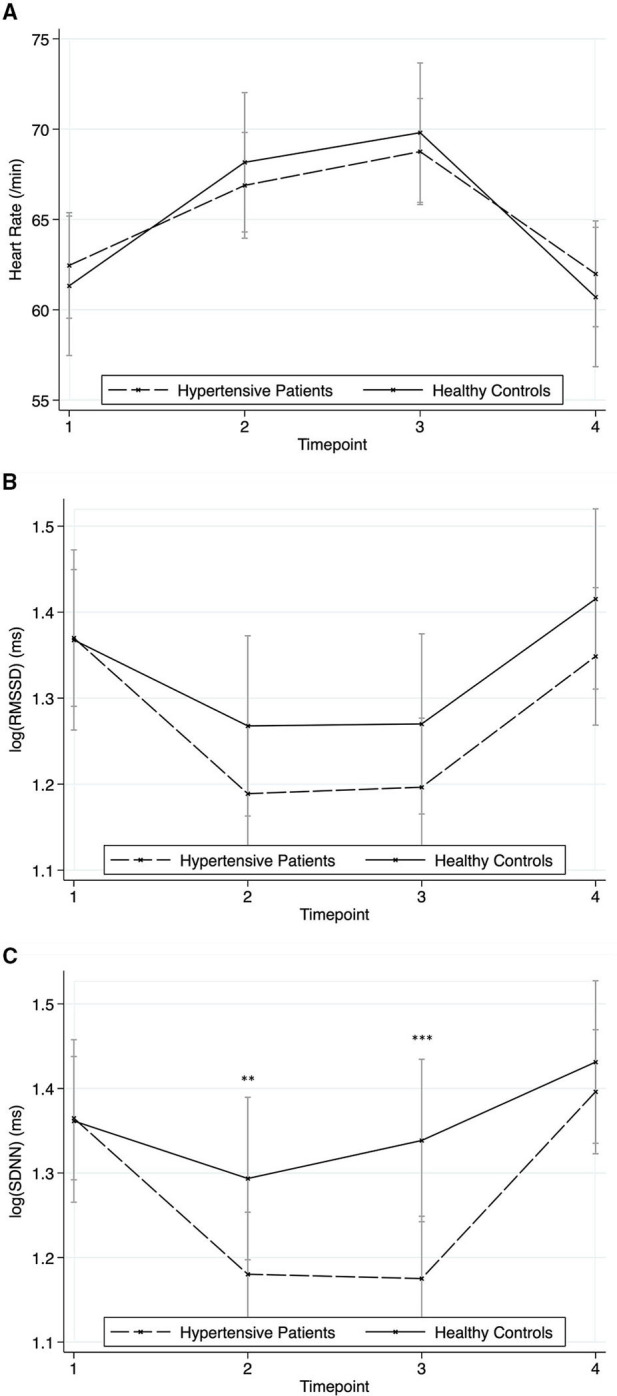
Responses in heart rate and heart rate variability to the stressors for hypertensive patients and healthy controls (mean ± SD). Timepoint 1: rest; timepoint 2: Attachment Interview (Adult Attachment Projective Picture System); timepoint 3: Separation Recall; timepoint 4: recovery. **(A)** Heart Rate. **(B)** Root mean square of successive differences (RMSSD). **(C)** Standard deviation of normal to normal intervals (SDNN). Asterisks indicate significant group*time differences related to healthy controls at reference timepoint 1 (**p < 0.01; ***p < 0.001).

HR reacted significantly to the stressors (time effect: chi2 = 260.04, *p* < 0.001) without a significant group effect (chi2 = 0.0, *p* = 0.993) or group^*^time-effect (chi2 = 7.69, *p* = 0.053) (Figure 4A). The results were similar for RMSSD (time effect: chi2 = 82.10, *p* < 0.001; group effect: chi2 = 0.69, *p* = 0.406; group^*^time-effect: chi2 = 4.25, *p* = 0.236) (Figure 4B). In contrast, results for SDNN showed a significant group^*^time-effect (chi2 = 18.05, *p* < 0.001) with hypertensives showing lower values at time point two (*z* = 2.70, *p* = 0.007) and three (*z* = 3.84, *p* < 0.001), further a significant time effect (chi2 = 91.81, *p* < 0.001) and no group differences (chi2 = 1.69, *p* = 0.194) (Figure 4C).

### Hypothalamic-Pituitary-Adrenal Axis

**Figure 5:**
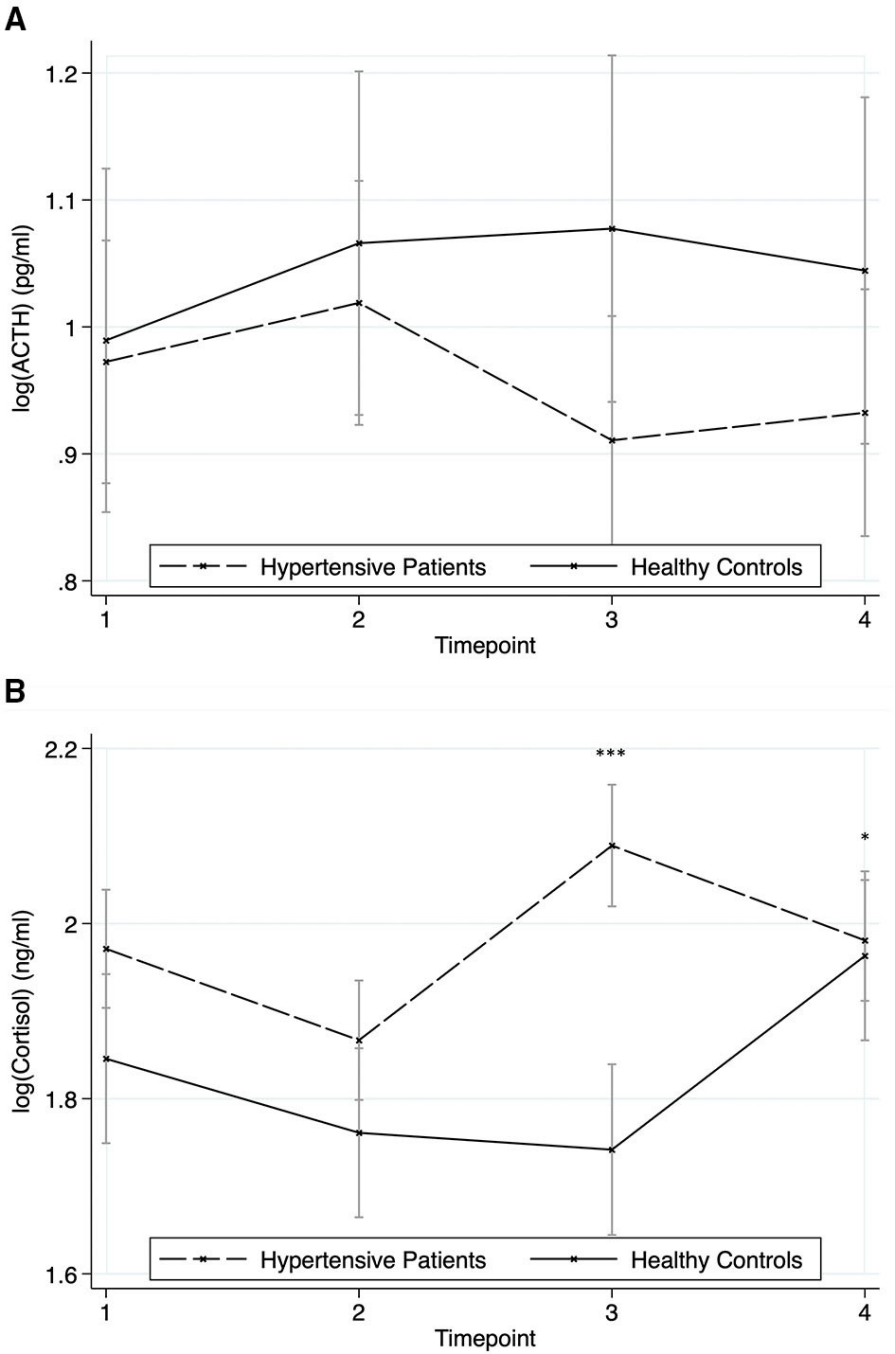
Responses of the hypothalamic-pituitary-adrenal axis to the stressors for hypertensive patients and healthy controls (mean ± SD). Timepoint 1: rest; timepoint 2: Attachment Interview (Adult Attachment Projective Picture System); timepoint 3: Separation Recall; timepoint 4: recovery. **(A)** ACTH. **(B)** Cortisol. Asterisks indicate significant group*time differences related to healthy controls at reference timepoint 1 (*p < 0.05; ***p < 0.001).

Figures 5A,B show the response of ACTH and cortisol. Neither group (chi2 = 1.25, *p* = 0.265) nor time (chi2 = 3.57, *p* = 0.312) nor group^*^time-effect (chi2 = 4.98, *p* = 0.173) were significant for ACTH. In contrast, post-estimation of contrasts revealed for cortisol next to the expected significant time effect (chi2 = 38.66, *p* < 0.001) a significant group effect (chi2 = 7.48, *p* = 0.006) with higher values in hypertensive patients and a significant group^*^time-effect (chi2 = 42.76, *p* < 0.001) with hypertensives showing an earlier peak at time point 3 (*z* = −4.3, *p* < 0.001), while the cortisol levels of healthy individuals rose at time point four (*z* = 2.1, *p* = 0.036).

### Catecholamines

**Figure 6:**
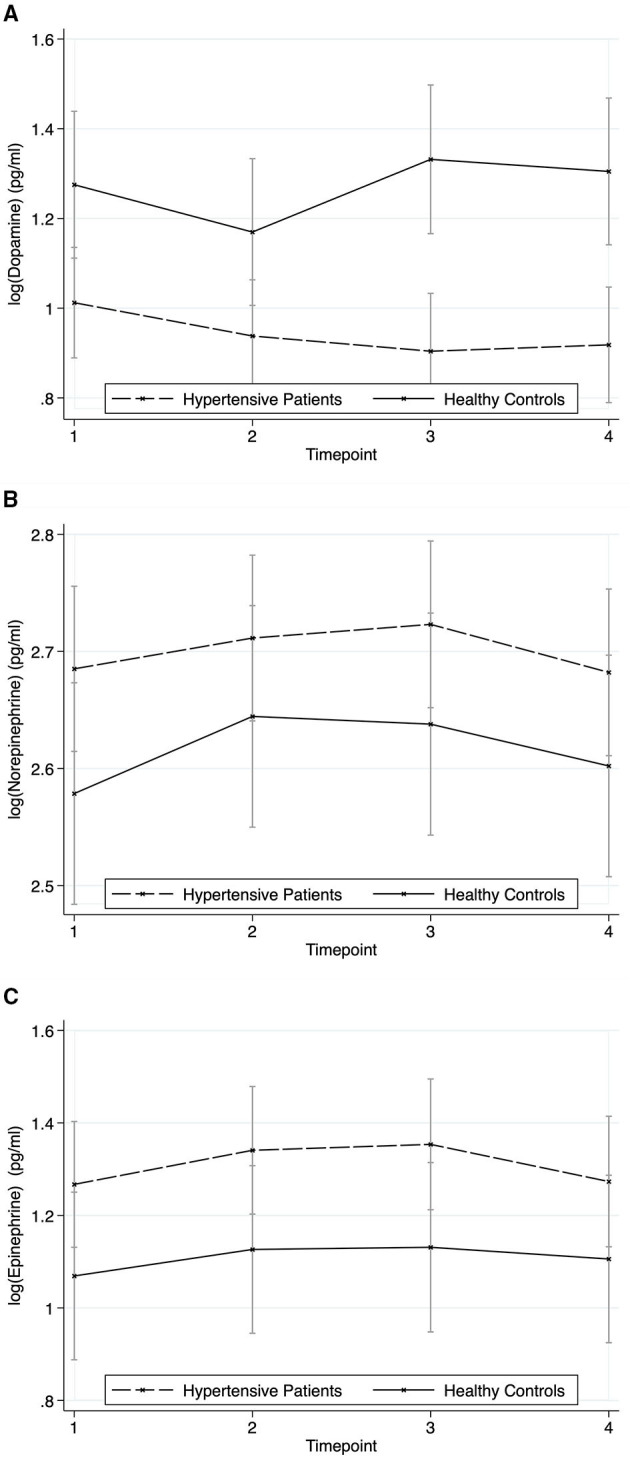
Responses of Catecholamines to the stressors for hypertensive patients and healthy controls (mean ± SD). Timepoint 1: rest; timepoint 2: Attachment Interview (Adult Attachment Projective Picture System); timepoint 3: Separation Recall; timepoint 4: recovery. **(A)** Dopamine. **(B)** Norepinephrine. **(C)** Epinephrine.

Post-estimation of contrasts from linear mixed-effects regression model for dopamine showed a significant group effect with hypertensive patients showing lower levels of dopamine than healthy controls (Figure 6A), but neither a time nor a group^*^time-effect (group effect: chi2 = 15.50, *p* < 0.001; time effect: chi2 = 2.61, *p* = 0.455; group^*^time effect: chi2 = 3.95, *p* = 0.267). Norepinephrine reacted significantly to the stressors (chi2 = 21.17, *p* < 0.001) without a significant group effect (chi2 = 1.87, *p* = 0.172) and without a group^*^time-effect (chi2 = 2.40, *p* = 0.493) (Figure 6B). The results for epinephrine were similar to dopamine, but with higher levels of epinephrine in patients with hypertension (group effect: chi2 = 4.04, *p* = 0.045; time effect: chi2 = 2.36, *p* = 0.502; group^*^time effect: chi2 = 0.27, *p* = 0.965) (Figure 6C).

### Arterial Stiffness

**Figure 7:**
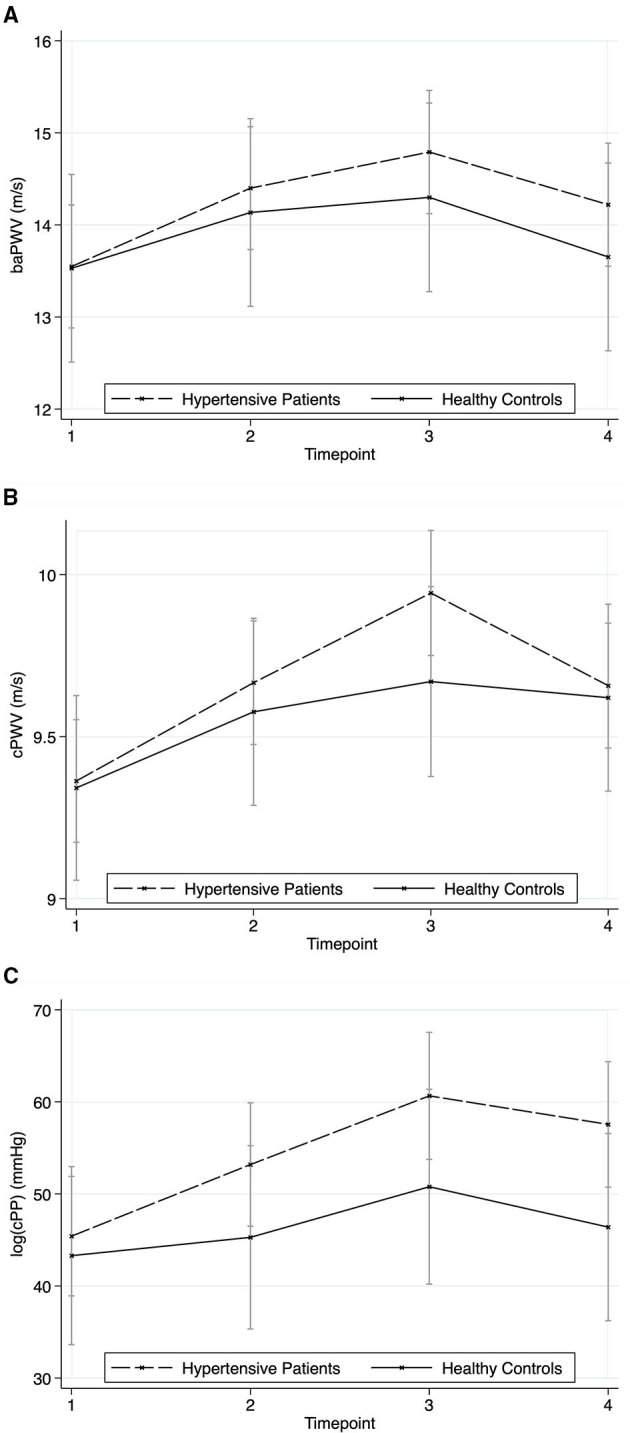
Responses in arterial stiffness to the stressors for hypertensive patients and healthy controls (mean ± SD). Timepoint 1: rest; timepoint 2: Attachment Interview (Adult Attachment Projective Picture System); timepoint 3: Separation Recall; timepoint 4: recovery. **(A)** Brachial-Ancle Pulse Wave Velocity. **(B)** Central Pulse Wave Velocity. **(C)** Central Pulse Pressure.

Figure 7 shows the results of baPWV (Figure 7A), cPWV (Figure 7B), and cPP (Figure 7C). All three parameters showed a significant time effect (chi2 = 34.49 for baPWV, chi2 = 67.02 for cPWV, chi2 = 18.43 for cPP, all *p* < 0.001) without significant group (chi2 = 0.30, *p* = 0.585; chi2 = 0.38, *p* = 0.540; chi2 = 1.43; *p* = 0.232) or group^*^time effects (chi2 = 2.84, *p* = 0.418; chi2 = 5.83, *p* = 0.120; chi2 = 1.39; *p* = 0.709).

## Discussion

We showed that medicated subjects with hypertension still show a heightened stress response to attachment/interpersonal-focused stressors with differences in the CV, hypothalamic-pituitary-adrenal (HPA) axis and autonomic reactivity compared to healthy subjects. This heightened reactivity might contribute to disease risk in patients with hypertension even though in rest, BP values might be within the normal range. It might represent the pathophysiological link for the finding of Kivimäki et al. that in men with cardiometabolic disease, job strain contributes significantly to risk of death, even in the group who had achieved treatment targets, while no risk was found in men without cardiometabolic disease (27).

The relation between laboratory stress and ambulatory blood pressure levels has been shown (28, 29). There is even evidence that the response to specific real life stressors is higher obtained in real life than in the laboratory (30).

The attachment-related stressors AAP and SR provoked a significant response of the CV system (BP, HR), the ANS, norepinephrine, and the HPA axis (cortisol). For the SR, this has already been shown regarding HR and BP (19).

There were no significant differences for SBP and DBP between hypertensive and healthy subjects and no significant group effects for BP. Therefore, we conclude that our study population was well-treated with regard to resting BP.

Still, hypertensives showed a steeper rise in blood pressure. We suppose that this is at least partly caused by a hyperreactive sympathetic nervous system (SNS) which we did not assess directly in our study but indirectly via HRV measurement. HRV represents the activity of the ANS and combines sympathetic and parasympathetic activity. Though there is no parameter representing mainly SNS activity, RMSSD is supposed to represent mainly vagal activity. Our results show an enhanced SDNN decline in hypertensive subjects compared to healthy ones while no such differences can be found regarding RMSSD. Therefore, we conclude that SNS activation is a major part of the heightened SDNN response next to vagal withdrawal. This is not supported by the norepinephrine response which was not enhanced in hypertensive subjects. However, there is evidence that norepinephrine in plasma does not reliably reflect SNS activity in general and especially not regional (15). Still, epinephrine levels were higher in hypertensive patients than in controls, a finding also reflected in other studies (11), which might reflect a higher systemic SNS tone. Our finding that levels of Dopamine which has natriuretic and vasodilating effects (31) were lower in hypertensive subjects than in controls is in line with the proposed imbalance of factors regulating sodium retention and vascular tone in essential hypertension (32).

Our results show that BP response is enhanced in hypertensives while HR response does not differ. An explanation might be that beta-blocking medication taken by 78% of the patients is effective in reducing HR and has a reducing, thus improving arterial stiffness (PWV) (33, 34), while BP response is still hyperreactive, i.e., this response is not blocked effectively. This would be in line with the results of Rothwell et al., who showed a higher BP variability in patients treated with beta-blocker compared with calcium channel blocker, which correlated with stroke risk (4).

Cortisol showed a significant rise in response to the stressor, while the rise in ACTH was not significant. This is not surprising, as the translation from ACTH to cortisol is not linear. Diurnal rhythms show a 5-fold higher rise in Cortisol than in ACTH. This is due to signal enhancement of ACTH via a risen ACTH-receptor-sensitivity, which can be SNS-mediated, and via a diurnal intra-adrenal regulation of the activity of enzymes synthesizing cortisol. Sympathetic innervation of the adrenal medulla, for instance, via the splanchnic nerve is known to play a critical role in modulating the diurnal adrenocortical sensitivity to ACTH (35, 36). The cortisol response to the stressor in our sample differed between healthy and hypertensive participants. While healthy controls showed a small, non-significant rise of ACTH in response to the stressors with a consecutive, physiologically delayed rise in cortisol, hypertensive patients responded faster with a steeper rise of cortisol levels. While Hamer and Steptoe found a higher cortisol response in individuals that later developed hypertension to those who did not in response to a mental stressor (10), this was not shown by Nyklicek in a study of hypertensive vs. healthy individuals in salivary cortisol levels (12). Wirtz found differences between healthy and hypertensive patients in terms of an attenuated cortisol awakening response and a suppressed feedback sensitivity after dexamethasone suppression (37). An altered HPA axis in hypertension might substantially contribute to the development of atherosclerosis via inflammation (38). In rats, both candesartan and ramipril attenuate Cortisol and ACTH response to CRH-stimulation (14). This finding was replicated in humans with diabetes (39). Though almost all of the hypertensive subjects in our study were on an ACE-inhibitor or an AT1-antagonist and the ACTH response seemed to be lower in hypertensive subjects than in controls, the cortisol response was not. This needs further investigation.

### Limitations

The study population might not be representative for subjects with hypertension in general as most of them already developed CV disease and took several antihypertensive drugs. Different kind of drugs have different effects on the physiological systems, and especially Dopamine and Epinephrine are affected by antihypertensive agents. However, due to small numbers and multiple combinations, we could not conduct separate analyses considering the different drugs. Therefore, the effects of some drugs may be masked. Also, we do not know the physiological response of the participants without taking drugs, so we do not know the net effect of the drugs. With consideration of the number of drugs and diagnoses, a cessation of drugs would not have been ethically justifiable. Further, we did not assess BP at home nor by a 24 h-measurement, so we can conclude adequacy of medication only by resting BP during the experiment.

The sample of hypertensive patients differed significantly from the healthy controls regarding age and BMI, both known risk factors for hypertension. We included them in our analyses as covariates. However, a subsample of hypertensive patients of age and BMI comparable to the healthy controls showed similar results with no deviances regarding significant effects.

Regarding the stressor, we neither included a solely cognitive mental stressor like a mental arithmetic, nor did we include a physical stressor. Thus, we cannot compare the stress response between cognitive mental, attachment-related mental, and physical stress.

It would have been desirable to directly measure neural activity of the SNS. Unfortunately, this was exceeding our resources. Further, the Renin-Angiotensin-Aldosteron-system is also not included in our parameters, which emerges to be another important stress axis (40).

## Conclusion

All in all, the response of the CV system, the ANS and the HPA axis is heightened in medicated subjects with hypertension in response to attachment-focused stressors compared to healthy subjects, though resting measurements do not differ between the groups. We suppose that the remaining hyper-reactivity even with sufficient antihypertensive medication still poses a substantial risk for affected patients. Sufficient antihypertensive treatment not only under ambulatory blood pressure control but by including stress-related measurements should be considered.

## Data Availability Statement

The raw data supporting the conclusions of this article will be made available by the corresponding author, EB, upon reasonable request without undue reservation.

## Ethics Statement

The studies involving human participants were reviewed and approved by ethical review board of Ulm University. The patients/participants provided their written informed consent to participate in this study.

## Author Contributions

EB designed the study, supervised data acquisition and conduction of the experiments, interpreted the data, and drafted the manuscript. MJ performed the statistical analyses and interpreted the data. SR and DL performed the measurement of ACTH and cortisol and interpreted part of the data. AS-T and SE supervised the PWV and PP measurements and interpreted part of the data. HG contributed to the conception of the experimental setting and critically revised the manuscript for important intellectual content. AF, JK, and SC planned and conducted the experiments and data acquisition, interpreted the data, and critically revised the manuscript for important intellectual content. CW contributed to the conception of the experimental setting, supervised the research project, and critically revised the manuscript for important intellectual content. All authors contributed to the article and approved the submitted version.

## Conflict of Interest

SE is employed at the moment by Economic Research and Policy Consultancy AG. This employment took place after his contribution to the manuscript (see Author Contributions) and is not related to any content of the experiment or the manuscript. The remaining authors declare that the research was conducted in the absence of any commercial or financial relationships that could be construed as a potential conflict of interest.

## Publisher's Note

All claims expressed in this article are solely those of the authors and do not necessarily represent those of their affiliated organizations, or those of the publisher, the editors and the reviewers. Any product that may be evaluated in this article, or claim that may be made by its manufacturer, is not guaranteed or endorsed by the publisher.

## Supplementary Material

Supplementary Material 1 Linear mixed-effects regression model for stress parameter reactivity: complete tables. Linear mixed-effects regression model with random intercepts. Covariates included in the models: age, body mass index. Time was level 1, the individual level (Persons) were on level 2. A two-way-interaction between group (Healthy vs. Hypertensive) and time (rest [1], AAP [2], SR [3], recovery [4]) was modeled using restricted maximum likelihood. Significance level: p < 0.05. df, degrees of freedom; bmi, body mass index; sbp, systolic blood pressure; dbp, diastolic blood pressure; HR, heart rate; n, normalized (with log-transformation); Cort, Cortisol; SDNN, standard deviation of normal to normal R-R intervals; RMSSD, root mean square of successive differences; ACTH, adreno-corticotrope hormone; baPWV, brachial-ankle pulse wave velocity; cPWV, central pulse wave velocity; cPP, central pulse pressure.
